# Differential effects of ethanol on behavior and GABA_A_ receptor expression in adult zebrafish (*Danio rerio*) with alternative stress coping styles

**DOI:** 10.1038/s41598-020-69980-2

**Published:** 2020-08-04

**Authors:** Alexander C. Goodman, Ryan Y. Wong

**Affiliations:** Department of Biology, University of Nebraska at Omaha, Omaha, NE 68182 USA

**Keywords:** Genetics of the nervous system, Ion channels in the nervous system, Molecular neuroscience

## Abstract

Variation in stress responses between individuals are linked to factors ranging from stress coping styles to sensitivity of neurotransmitter systems. Many anxiolytic compounds (e.g. ethanol) can increase stressor engagement through modulation of neurotransmitter systems and are used to investigate stress response mechanisms. There are two alternative suites of correlated behavioral and physiological responses to stressors (stress coping styles) that differ in exploration tendencies: proactive and reactive stress coping styles. By chronically treating individuals differing in stress coping style with ethanol, a GABA-acting drug, we assessed the role of the GABAergic system on the behavioral stress response. Specifically, we investigated resulting changes in stress-related behavior (i.e. exploratory behavior) and whole-brain GABA_A_ receptor subunits (*gabra1*, *gabra2*, *gabrd*, & *gabrg2*) in response to a novelty stressor. We found that ethanol-treated proactive individuals showed lower stress-related behaviors than their reactive counterparts. Proactive individuals showed significantly higher expression of *gabra1*, *gabra2,* and *gabrg2* compared to reactive individuals and ethanol treatment resulted in upregulation of *gabra1* and *gabrg2* in both stress coping styles. These results suggest that impacts of ethanol on stress-related behaviors vary by stress coping style and that expression of select GABA_A_ receptor subunits may be one of the underlying mechanisms.

## Introduction

While an organism’s stress response is essential to its survival, not all conspecifics exhibit similar responses and often differ both behaviorally and physiologically^1–5^. Upon perception of a stressor the nervous system simultaneously stimulates the sympathetic adrenal medullary and the hypothalamic–pituitary–adrenal pathways within mammals that rapidly lead to changes in behavior and the endocrine system. An individual’s behavioral and physiological stress responses are often correlated and are consistent across contexts. Throughout many taxa there exists two alternative correlated suites of behavioral and physiological responses to stressors known as the proactive and reactive stress coping styles^2,3,5–7^. In response to novelty, proactive individuals actively engage stressors and characteristically exhibit a lower whole-body cortisol response compared to reactive individuals^2,3,5,8–11^. Additionally, proactive and reactive individuals differ in expression of key neurotransmitter receptors related to stress and anxiety, such as serotonin, dopamine, and GABA (γ-amino butyric acid) receptors^2,3,12,13^. Drugs designed to target such systems are often employed to study a neurotransmitter’s influence on stress-related behaviors^14–16^. Therefore, pharmaceuticals can be used to investigate underlying differences in the molecular mechanisms between stress coping styles. Specifically, measuring different molecular responses to behavioral-altering anxiolytics or anxiogenics can provide insight on the underlying mechanisms of these individual differences and ultimately stress and anxiety.

Dysregulation of the GABAergic, the serotoninergic, and the glutamatergic systems often contribute to a disproportional behavioral stress response^14,17^, which, if sustained over an extended period of time, can be classified as an anxiety disorder^18,19^. GABAergic system dysfunction is thought to contribute to the underlying etiology of anxiety-related disorders^20,21^. GABA_A_ receptor (GABA_A_R) agonists, such as ethanol, allow for positive modulation of the GABAergic system to produce an anxiolytic response, while antagonists result in an anxiogenic response in rodents (*Rattus norvegicus*, *Mus musculus*) and zebrafish (*Danio rerio*) ^16,17,22–31^. The GABA_A_R itself is a pentamer composed of any combination of the α-, β-, γ-, δ-, ε-, and θ-subunits and each has their own respective variants (α_1_–α_6_, β_1_–β_3_, γ_1_–γ_3_, ρ_1_–ρ_3_, δ, ε, θ)^32^. GABA-acting drugs influence the expression of the protein subunits that make up the receptor subtype^32,33^. For example, rodents exposed to GABA_A_ agonists show an increase in expression of the α_1_-, α_2_-, and δ-subunits of the GABA_A_R, while expression of the γ_2_-subunit decreases^34–37^. Studies utilizing zebrafish similarly show that ethanol administration produces anxiolytic behavioral effects^14,23,30,31,38,39^. In zebrafish, there are baseline differences in mRNA expression of both GABA_A_ and GABA_B_ receptors between the two stress coping styles^13^. How GABA-acting drugs differentially influence both behavior and physiology between them, however, is not understood.

Zebrafish are widely used to understand the effects of pharmaceuticals on stress and anxiety-related behaviors and physiology due to their conserved behavioral, neuroanatomical, pharmacological, and transcriptional stress responses with mammals and other species^14–16,31,40–44^. Many studies have examined the anxiogenic and anxiolytic impacts of pharmaceuticals and developed a variety of behavioral assays to measure stress and anxiety^14,16,41,45–48^. For example one assay used to quantify stress-related behaviors is the Novel Tank Diving Test (NTDT), which measures a subject’s level of vertical exploration as a behavioral proxy for stress^10,31,45,49,50^. In the NTDT there is an inverse relationship between stress levels and depth preference^10,31,45,49,50^. Several studies have also validated the NTDT as a model to study anxiolytic compounds (e.g. GABA acting drugs)^51–53^. Many studies have focused on acute effects of ethanol but relatively less is known on effects of chronic treatment^14,30,49,54–56^.

Of note both wild and laboratory strains of zebrafish show the proactive and reactive stress coping styles^5,6^. These coping styles in zebrafish display differences in genetic backgrounds, behavior and neuroendocrine responses to stressors that are consistent with what has been documented in birds and mammals^14,57,58^. Using artificial selection, we previously generated two lines of zebrafish (low stationary behavior, LSB; high stationary behavior, HSB) that show consistency with the proactive and reactive stress coping styles. More specifically, the LSB and HSB lines show consistent differences in stress-related behaviors across multiple behavioral assays, morphology and escape performance, whole-brain transcriptome profiles, cognitive performances, and endocrine responses characteristic of the proactive and reactive stress coping styles, respectively^5–7,10,11,59–63^. Only recently are studies beginning to demonstrate the roles of synaptic plasticity and neurotransmitter system regulation in facilitating the display of alternative stress coping styles in zebrafish^5,7,13,59,62,64^. However, the differential impact of GABA-acting drugs (e.g. ethanol) on behavior and the GABA system between stress coping styles is just beginning to be explored^55,56^.

In this study, we assessed the effects of ethanol treatment on stress-related behavior and GABA_A_R subunit gene expression in two zebrafish lines selectively bred to display the proactive and reactive stress coping styles. Specifically, we quantified exploratory behavior using the NTDT and expression of four genes encoding for the α_1_-, α_2_-, δ-, and γ_2_-subunits of the GABA_A_R (*gabra1, gabra2, gadrd,* and *gabrg2,* respectively^65^. These particular subunits were chosen as they are found in relatively high abundance in the GABA_A_R^32,35^, and previous studies in other species suggest the expression of these subunits is altered by GABA-acting drugs^34–37^. We hypothesized that chronic ethanol treatment will reduce stress-related behaviors in both lines of zebrafish with a greater anxiolytic response for the reactive line. Additionally, based on previous literature we predicted to see an increase in mRNA expression of α_1_-, α_2_-, δ-subunits and decrease expression of the γ_2_-subunit for both lines but the magnitude of the effect would be greater in the reactive line^34–37^. Understanding how a GABA_A_R agonist impacts GABA neurotransmission between the two coping styles will give insight into one mechanism that may explain differences in their stress and anxiety-related behavioral responses.

## Results

### Identifying an ethanol treatment duration and concentration that produces an anxiolytic effect across lines

**Figure 1:**
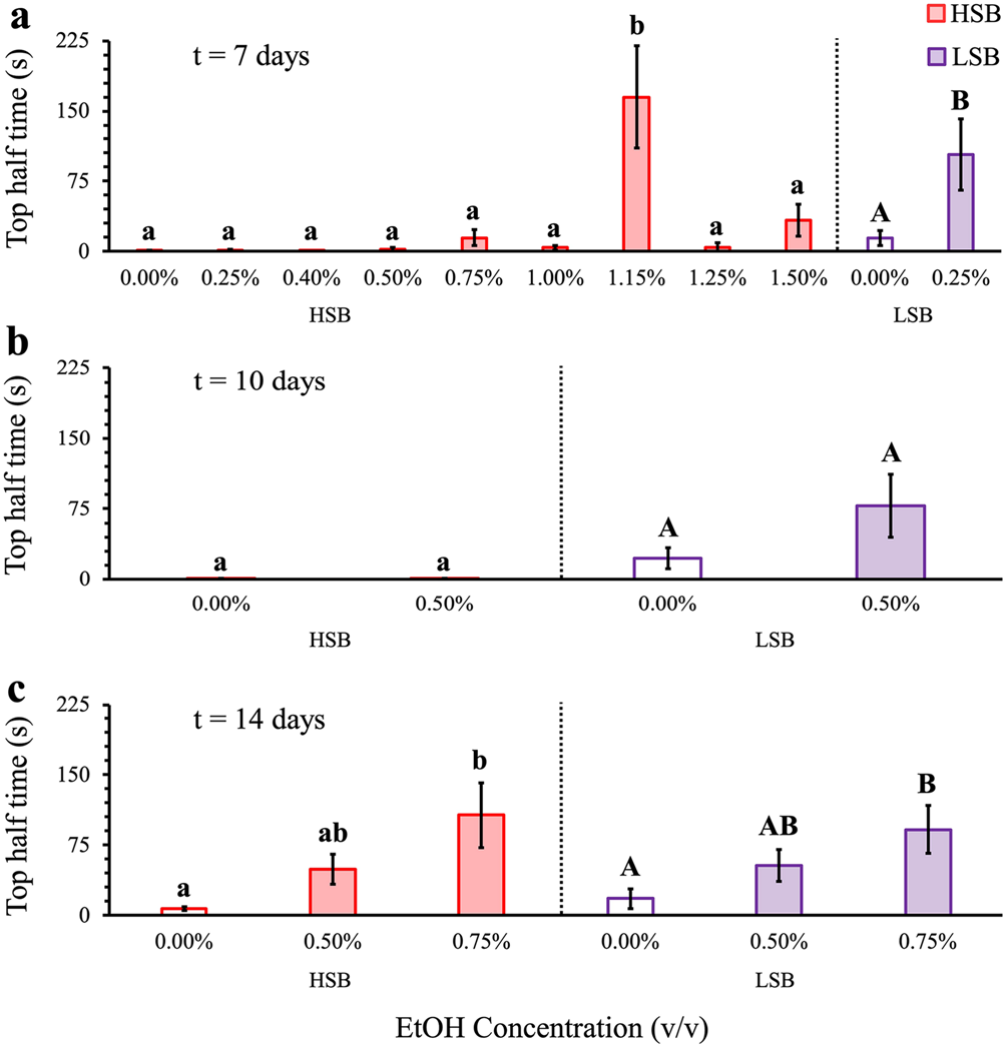
Dose response analysis of ethanol concentration on time spent in the top half of the tank during NTDT. Measured time spent in the top half of the tank after (**a**) 7, (**b**) 10, or (**c**) 14 days of treatment. Control groups are represented by unfilled in bars, while ethanol-treated groups are represented by filled bars. HSB and LSB are red and purple, respectively. Data shown are mean ± 1 SEM. Individual differences within the HSB line are indicated by lower case letters, while differences within the LSB line are indicated by upper case letters.

To find a biologically relevant dose and treatment length applicable to both HSB and LSB fish, we tested durations from 7 days (0.25%, 0.4%, 0.5%, 0.75%, 1%, 1.15%, 1.25%, and 1.5% ethanol), 10 days (0.5% ethanol), and 14 days (0.5% and 0.75% ethanol) (Fig. 1, Supplementary Table S1). There were significant main effects of 0.75% ethanol concentration on time spent in the top half of the tank for both the HSB and LSB lines at the 14-day duration [HSB: Wald *χ*^2^(2) = 12.338, *p* = 4.43 × 10^–4^; LSB: Wald *χ*^2^(2) = 8.707, *p* = 0.003]. Examination of simple main effects revealed fish treated with 0.75% ethanol concentration showed an increase in time spent in the top half of the tank compared to 0.0% concentration for both the HSB and LSB line (HSB: *p* = 1.70 × 10^–5^; LSB: *p* = 0.003) with no drug-impaired locomotion. Therefore, we selected the 0.75% ethanol for two weeks as the treatment regime for this study. Full model results are presented in Supplementary Table S1.

### Ethanol-treatment increases exploration for both lines

**Figure 2:**
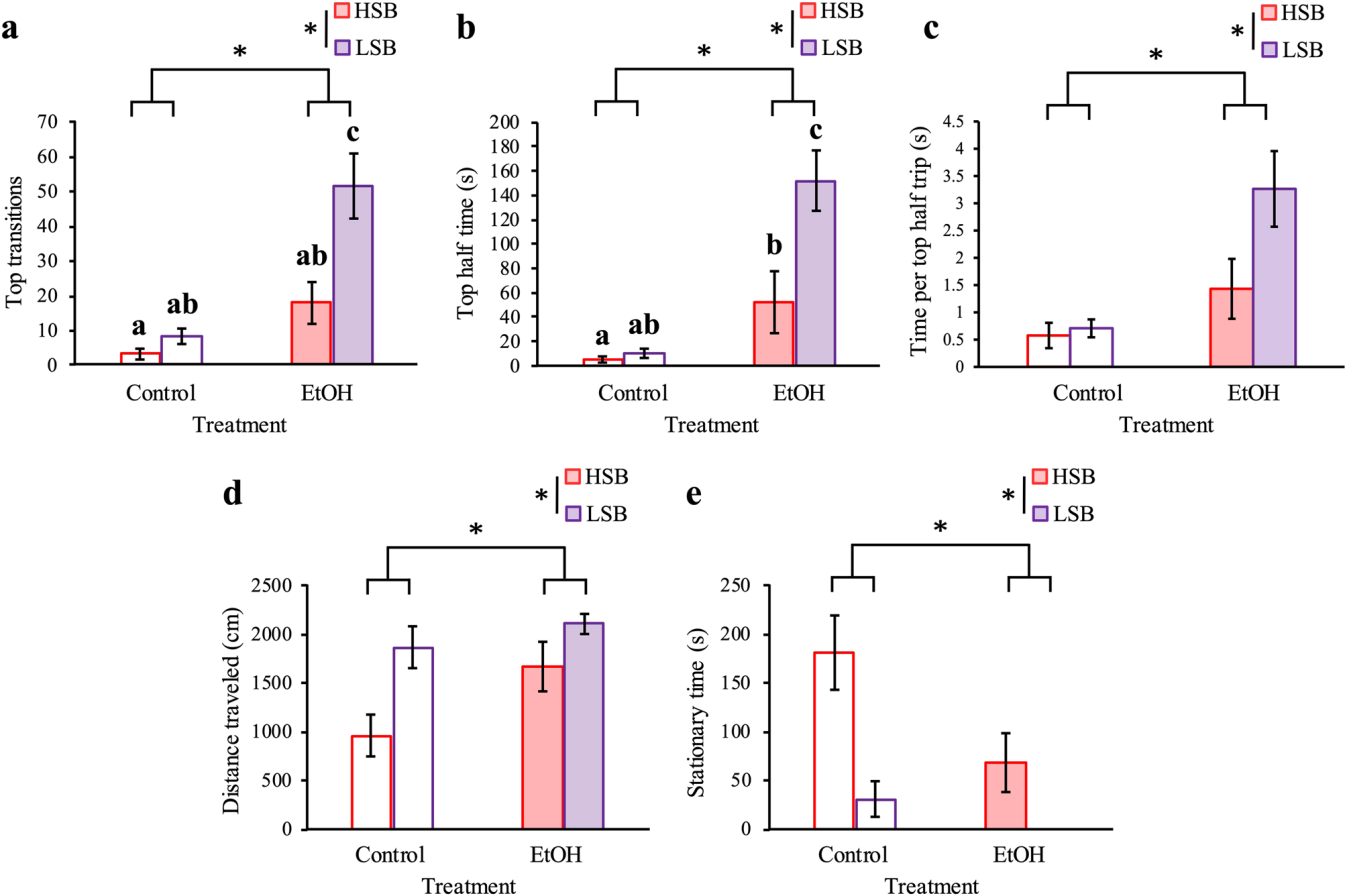
Differentiated ethanol treatment effect on stress-related behaviors between lines with no effect on locomotion. We measured top transitions (**a**), time in top half of the tank (**b**), average time spent in top half per trip (**c**), distance traveled (**d**), and stationary time (**e**) for each treatment group. Control groups are represented by unfilled in bars, while ethanol-treated groups are represented by filled bars. HSB and LSB are red and purple, respectively. Data shown are mean ± 1 SEM. Significant line and treatment differences are indicated by an asterisk (*p* ≤ 0.05), while differences between groups are indicated by different lower-case letters. The number of subjects tested in each group are as follows: 17 HSB control, 17 LSB control, 15 HSB EtOH, 16 LSB EtOH.

There were significant main effects of line (HSB, LSB) on number of top transitions [Wald *χ*^2^(1) = 12.579, *p* = 3.90 × 10^–4^], time spent in the top half of the tank [Wald *χ*^2^(1) = 10.215, *p* = 0.001], and time per trip to top half [Wald *χ*^2^(1) = 5.045, *p* = 0.025]. LSB fish transitioned to the top half of the tank (*p* = 3.90 × 10^–4^; Fig. 2a), spent more time in the top half of the tank (*p* = 0.001; Fig. 2b; Supplementary Fig. S1), and spent longer time per trip to the top half (*p* = 0.025; Fig. 2c) than HSB fish. There were also significant main effects of treatment (ethanol, control) on top transitions [Wald *χ*^2^(1) = 28.054, *p* = 1.18 × 10^–7^], time spent in the top half of the tank [Wald *χ*^2^(1) = 32.659, *p* = 1.10 × 10^–8^], and time per trip to top half [Wald *χ*^2^(1) = 15.227, *p* = 9.53 × 10^–5^]. Ethanol-treated fish transitioned to the top half of the tank (*p* = 1.18 × 10^–7^), spent significantly more time in the top half of the tank (*p* = 1.10 × 10^–8^), and spent longer time per trip to the top half of the tank (*p* = 9.53 × 10^–5^) than control fish. There was a significant line by treatment interaction effect for transitions to the top half of the tank [Wald *χ*^2^(1) = 6.788, *p* = 0.009] and time spent in the top half of the tank [Wald *χ*^2^(1) = 8.182, *p* = 0.004]. There was a trend for a line by treatment interaction effect for time per trip to top half [Wald *χ*^2^(1) = 3.784, *p* = 0.052]. Ethanol-treated LSB fish exhibited the most top transitions compared to the control HSB (*p* = 2.85 × 10^–10^), control LSB (*p* = 1.75 × 10^–8^), and ethanol-treated HSB fish (*p* = 2.12 × 10^–5^). This pattern was also found for time spent in the top half with ethanol-treated LSB exhibiting the most time spent in the top half of the tank compared to control HSB (*p* = 2.09 × 10^–10^), control LSB (*p* = 9.63 × 10^–10^), and ethanol-treated HSB fish (*p* = 2.84 × 10^–5^). The LSB ethanol-treated fish averaged more time per trip to the top half of the tank than the control HSB (*p* = 1.16 × 10^–5^), control LSB (*p* = 3.05 × 10^–5^), and ethanol-treated HSB fish (*p* = 0.004). Full model results are presented in Supplementary Table S2.

### No impaired locomotion from ethanol-treatment for both lines

There were significant line effects for total distance swam [Wald *χ*^2^(1) = 11.378, *p* = 0.001] and stationary time [Wald *χ*^2^(1) = 18.173, *p* = 2.02 × 10^–5^]. LSB fish swam a significantly farther distance (*p* = 0.001; Fig. 2d) and spent significantly less time stationary (*p* = 2.02 × 10^–5^; Fig. 2e) than HSB fish. We also found significant treatment effects for total distance swam [Wald *χ*^2^(1) = 5.729, *p* = 0.016] and stationary time [Wald *χ*^2^(1) = 7.831, *p* = 0.005]. Ethanol-treated fish traveled farther (*p* = 0.016) and spent less time stationary (*p* = 0.005) than control fish. There were no significant line by treatment interaction effects for total distance traveled [Wald *χ*^2^(1) = 1.391, *p* = 0.238] or stationary time [Wald *χ*^2^(1) = 2.639, *p* = 0.104]. Full model results are presented in Supplementary Table S2.

When examining changes in locomotion within a trial there was a significant effect of trial time for both lines on distanced traveled [HSB: Wald *χ*^2^(1) = 23.359, *p* = 2.88 × 10^–4^; LSB: Wald *χ*^2^(1) = 45.354, *p* = 1.23 × 10^–8^] and transitions to the top half. [HSB: Wald *χ*^2^(1) = 14.059, *p* = 0.015; LSB: Wald *χ*^2^(1) = 14.000, *p* = 0.016; Supplementary Fig. S2]. Transitions increased as the trial progressed, which suggests that habituation occurred to the testing chamber during the trial. There were no significant main effects of trial time on time in top half [HSB: Wald *χ*^2^(1) = 8.564, *p* = 0.128; LSB: Wald *χ*^2^(1) = 1.158, *p* = 0.949], time per trip to the top half [HSB: Wald *χ*^2^(1) = 4.469, *p* = 0.484; LSB: Wald *χ*^2^(1) = 5.112, *p* = 0.402], or stationary time [HSB: Wald *χ*^2^(1) = 2.675, *p* = 0.750; LSB: Wald *χ*^2^(1) = 7.083, *p* = 0.215] for either line (Supplementary Fig. S2). The only significant treatment by time interaction effects were seen on number of top transitions [Wald *χ*^2^(1) = 12.211, *p* = 0.032] and time per trip to top half [Wald *χ*^2^(1) = 13.785, *p* = 0.017] in the LSB line. Full model results are presented in Supplementary Table S3.

### Ethanol-treatment increases expression of *gabra1* and *gabrg2*

**Figure 3:**
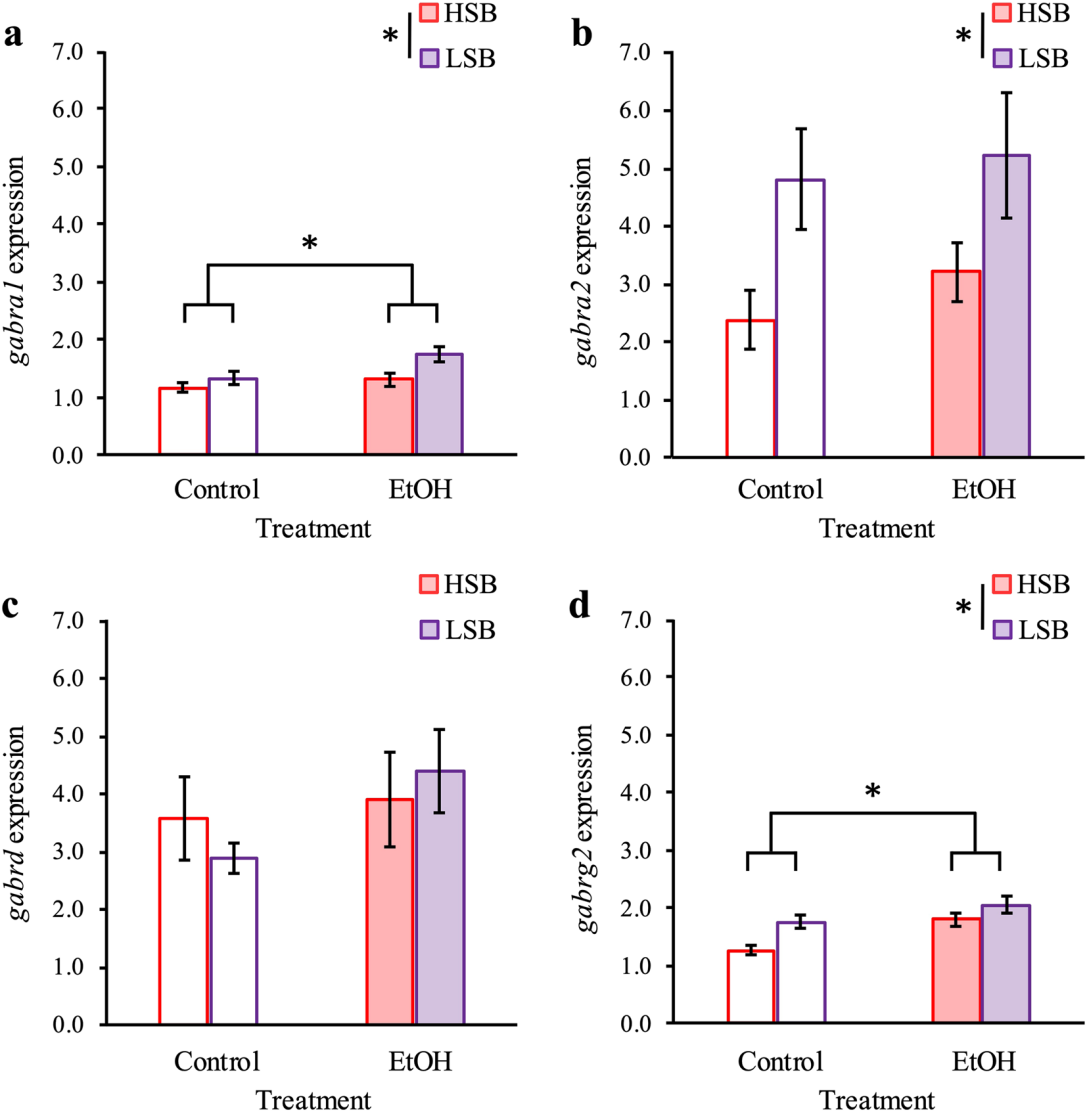
Effect of line and treatment on GABA_A_ receptor subunits. Normalized expression of *gabra1* (**a**), *gabra2* (**b**), *gabrd* (**c**), and *gabrg2* (**d**) for each treatment group following treatment. Control groups are represented by unfilled in bars, while ethanol-treated groups are represented by filled bars. HSB and LSB are red and purple, respectively. Data shown are mean ± 1 SEM. Significant differences are indicated by an asterisk (*p* ≤ 0.05). The number of subjects tested in each group are as follows: 17 HSB control, 18 LSB control, 17 HSB EtOH, 17 LSB EtOH.

We found significant main effects of line on expression of *gabra1* [Wald *χ*^2^(1) = 7.310, *p* = 0.007; Fig. 3a]*, gabra2* [Wald *χ*^2^(1) = 8.235, *p* = 0.004; Fig. 3b]*,* and *gabrg2* [Wald *χ*^2^(1) = 5.929, *p* = 0.015; Fig. 3d], but not *gabrd* [Wald *χ*^2^(1) = 0.023, *p* = 0.880; Fig. 3c]. The LSB fish showed higher expression of the *gabra1* (*p* = 0.007), *gabra2* (*p* = 0.004), and *gabrg2* (*p* = 0.015) than the HSB fish. There were significant main effects of treatment on expression of *gabra1* [Wald *χ*^2^(1) = 6.507, *p* = 0.011] and *gabrg2* [Wald *χ*^2^(1) = 7.220, *p* = 0.007] but not *gabra2* [Wald *χ*^2^(1) = 0.648, *p* = 0.421] or *gabrd* [Wald *χ*^2^(1) = 2.042, *p* = 0.153]. Ethanol-treated fish showed greater expression of *gabra1* (*p* = 0.011) and *gabrg2* (*p* = 0.007) than control fish. There were no significant line by treatment interaction effects for any of the four genes of interest [*gabra1*: Wald *χ*^2^(1) = 1.339, *p* = 0.247; *gabra2*: Wald *χ*^2^(1) = 0.073, *p* = 0.787; *gabrd*: Wald *χ*^2^(1) = 0.832, *p* = 0.362; *gabrg2*: Wald *χ*^2^(1) = 0.659, *p* = 0.417]. Full model results are presented in Supplementary Table S4.

## Discussion

GABA_A_R agonists, such as ethanol, produce an anxiolytic response across many taxa^14,17,22,23,29–31,66,67^. Through the use of these stress-reducing compounds, we can investigate the role of the GABAergic system in facilitating the expression of a stress coping style. In this study, we assessed both the behavioral and molecular responses of ethanol treatment between proactive (LSB) and reactive (HSB) lines of zebrafish. We found that while chronic ethanol treatment decreased stress-related behaviors in both lines, ethanol treatment had a greater anxiolytic effect on the LSB line. The differences in stress-related behavior are linked to differential GABA_A_R subunit expression between the lines (α_1_-, α_2_-, and γ_2_-subunits) or in response to ethanol treatment (α_1_-, and γ_2_-subunits). The results suggest molecular differences in the GABAergic neurotransmitter system contribute to the variation in stress-related behaviors between the two stress coping styles.

The anxiolytic behavioral response to ethanol in zebrafish is well documented^14,23,31,38,39^, but the effect of an individual’s stress coping style on the response to GABA_A_R agonists has only been investigated recently^55,56^. We predicted that treatment with a GABA_A_R agonist would have a greater anxiolytic effect on both stress-related behaviors and GABA_A_R subunit expression in the reactive stress coping style than the proactive stress coping style. As expected, we found that both the LSB (proactive) and HSB (reactive) lines significantly increased their exploration and locomotion with chronic ethanol treatment. There are at least two alternative interpretations of the behavioral changes due to ethanol treatment: anxiolytic or anxiogenic and hyperactive effect. Increased distance traveled and number of transitions to the top half are predicted to occur with both stress-induced hyperactivity and stress reduction (e.g. more motivation to explore). When considering time duration in the top half, if an anxiolytic and hyperactive effect occurred, then we would have expected decreased time in the top and shorter time per trip in the top relative to controls. However, ethanol treated fish spent significantly more time in the top half and longer time in the top half per trip, which suggests an anxiolytic effect of ethanol treatment. These behavioral displays are also consistent with other studies that administered anxiolytic and stress-reducing pharmaceuticals^14,30,31,39,68,69^. Furthermore, we and others have shown that zebrafish that spend more time in the upper half of the water column also showed significantly lower cortisol levels^10,45,70^. Altogether our results in conjunction with prior studies suggest ethanol treatment resulted in an anxiolytic effect rather than a hyperactive and anxiogenic effect.

Surprisingly, the proactive individuals showed a greater anxiolytic response than the reactive individuals. To our knowledge, only a couple of other studies have accounted for stress coping style when examining the anxiolytic effects of ethanol in zebrafish^55,56^. In these studies, acute ethanol treatment (60 min) resulted in a greater anxiolytic effect (fish spent more time in an area of the tank furthest from conspecifics, swan faster, and traveled further from the bottom half of the tank) on reactive fish, while proactive fish increased their stress-related behaviors^55,56^. We speculate the opposing observations between our studies could be due to differences in treatment length (60 min vs. 2 weeks), social stress buffering (social vs. isolation), and assignment of stress coping style (behavioral screen vs. selectively bred lines). Regardless, ethanol is known to have an anxiolytic effect and the behavioral results from the prior and current studies suggest that an individual’s stress coping style can modulate the magnitude of the effect.

We found that the LSB line of zebrafish showed the greatest increase in transitions to, time spent in the top half of the tank, and average top half trip time during the NTDT compared to the HSB line (Fig. 2a–c). This line-specific response can be seen in other zebrafish studies and also in rodents^5,6,10,55,56,68,69,71–74^. Laboratory lines of zebrafish require a higher concentration of ethanol to match exploratory behavior of wild-caught lines, while wild-caught lines exhibit abolishment of shoaling behavior at higher concentrations of ethanol^68,69,72^. Rodents selectively bred to exhibit diverging novelty-seeking behaviors show differing levels of responsiveness to ethanol^71,73,74^. Maintaining laboratory and selectively bred lines of animals simultaneously results in line-specific genetic backgrounds. For example, the HSB and LSB zebrafish lines used here show distinct whole-brain transcriptome profiles^13,63^, and divergent novelty-seeking rodent lines differ in neuropeptide gene expression relating to the dopaminergic system^71,74^. This suggests an individual’s behavioral response can be influenced by its genetic profile and underlying expression of neurotransmitters. Altogether our results show that differences in molecular mechanisms can contribute to the alternative behavioral stress-response between stress coping styles.

It is possible that the higher expression of α_1_-, α_2_-, and γ_2_-subunits GABA_A_R subunits we observed in this study in the proactive (LSB) zebrafish facilitated a greater anxiolytic response to ethanol treatment than in the reactive (HSB) zebrafish (Fig. 3a, b, d). In rodents, removal of the α_2_-subunit results in the abolishment of the anxiolytic effect for both ethanol and other benzodiazepines^75,76^, suggesting this is a critical subunit needed for ethanol’s anxiolytic effect. We hypothesize that higher expression of these subunits in our proactive line may allow for greater sensitivity of GABA_A_R ligands leading to a greater anxiolytic response.

In addition to being differentially expressed between the two lines, expression of the α_1_-, and γ_2_-subunits increased as a result of ethanol treatment (Fig. 3a, d). These results are consistent with previous studies in rodents where α_1_-subunit increased expression with ethanol treatment^34–37^, suggesting that ethanol-induced modulation of this subunit may be a conserved response across taxa. Prior studies examining the change in the γ_2_-subunit expression to ethanol treatment show conflicting information^77–79^. While our results are consistent with studies showing lower expression of this particular subunit decreases stress-related behaviors, other studies have shown increased expression similarly leading to a reduction in stress-related behaviors. It has been hypothesized that the γ_2_-subunit increases the overall responsiveness of the GABA neurotransmitter system^79,80^. Our results are consistent with this hypothesis as the proactive line showed higher expression of the γ_2_-subunit and had a greater change in the anxiolytic behavioral response from a GABA_A_R agonist (ethanol). Interestingly, knockouts of either the α_1_- or γ_2_-subunits do not abolish ethanol’s anxiolytic effect. Both wild type and α_1_-subunit knockout rodents display an anxiolytic response to GABA_A_R agonists, but rodents with the knockout display a greater decrease in anxiety-related behaviors, such as time spent in the open and number of open arm entries in the elevated plus maze^81–83^. Results of previous studies assessing γ_2_-subunit knockouts on stress-related behaviors are inconsistent. Some studies found partial knockout of this receptor subtype decreases exploratory behavior in an open field test (i.e. increasing anxiety)^77,78^, while a more recent study found complete knockout of the subunit in dopaminergic neurons increases exploratory behavior^79^. While removal of the α_1_- or γ_2_-subunits alters behavior in the rodent animal model, the anxiolytic effect of GABA_A_R agonist is still present regardless of the presence in the GABA_A_R. This suggests that the α_1_- and γ_2_-subunits are sufficient but not necessary for the anxiolytic response and their increased expression in the current study may have facilitated the reduction of stress-related behavioral displays in both lines.

Of note, we did not observe any significant line by treatment interaction effects on expression of any of the examined GABA_A_R subunits. It is possible that by looking at whole-brain expression levels, we masked brain-region specific responses that may have shown interaction effects. Published data suggest that within broad brain divisions implicated in regulating stress (e.g. telencephalon, diencephalon), α_1_-, α_2_-, and γ_2_-subunits of GABA_A_R showed similar expression across individuals in the telencephalon and olfactory bulbs, but were more variable within the diencephalon^84–86^. While studies have demonstrated differences in GABAergic neurons and GABA_A_R neurons across broad divisions of the zebrafish brain^84–86^, to our knowledge no study has mapped and quantified GABA_A_R distribution at the resolution of individual brain nuclei in zebrafish. Future studies comparing GABA_A_R expression within the network of brain regions regulating stress and anxiety-like behavior between alternative stress coping styles in zebrafish are needed. As the GABAergic system can be differentially modulated depending on length (acute vs chronic) of ethanol exposure^67,72,87^, we also cannot rule out the possibility that our results may change with acute ethanol exposure. Another possibility is that other GABA_A_R subunits that were not examined in this study could influence stress-related behaviors between stress coping styles. In rodents ethanol treatment alters expression of α_4_-, α_5_-, and γ_1_-subunits^34,88,89^. Finally, it is possible that the GABAergic system does not play a significant role in the differentiated anxiolytic behavioral effects of chronic ethanol exposure between stress coping styles in zebrafish. Rather, the anxiolytic effects could be mediated by another neurotransmitter system such as the dopaminergic or serotoninergic system. Prior studies in fish and rodents have documented that administration of ethanol and other anxiolytic compounds alter several neurotransmitter systems in addition to the target system^60,90–95^. Of note, a prior study showed that the proactive (LSB) line showed higher baseline expression of the dopamine receptor D2 compared to the reactive (HSB) line^13^. Given this receptor’s role in ethanol-induced activation of the mesolimbic dopaminergic reward pathway of the brain and drug-seeking and novelty exploration behaviors^96–98^, we speculate that the differences in the magnitude of the anxiolytic effects of chronic ethanol on behavior between the two stress coping style lines involve the dopaminergic system. Future studies are needed to assess the extent of ethanol effects on neurotransmitter systems beyond the GABA_A_ system between the two stress coping styles.

## Conclusions

In this study, we showed significant main effects of line on anxiety-related behaviors and GABA_A_R subunit expressions where individuals with the proactive (LSB line) stress coping style had lower anxiety-related behaviors and higher expression of the α_1_, α_2_, and γ_2_-subunits relative to reactive (HSB line) individuals. This demonstrates that variation in behavioral responses to a novelty stressor may be explained by differences in the GABAergic system (e.g. GABA_A_R subunit expression) between the two stress coping styles. Intriguingly, we observed a significant line by ethanol treatment interaction effects on stress and anxiety-related behaviors. Chronic ethanol treatment had a surprisingly greater anxiolytic effect on proactive individuals, which suggests that ethanol alters the underlying neuromolecular mechanisms in a coping style-specific manner. However, the lack of an interaction effect between line and treatment on any of the four measured GABA_A_R subunits leads us to speculate that the differences in the magnitude of effect between the lines induced by chronic ethanol treatment may be mediated by other GABA_A_R subunits or a neurotransmitter system other than the GABAergic system. More broadly, this study shows that differences in stress and anxiety-related behaviors between the proactive and reactive stress coping styles are due in part to differences in the GABAergic system, but any coping-style specific anxiolytic behavioral effects of chronic ethanol exposure likely involve other neurotransmitter systems.

## Methods

### Subjects

In this study, we used the high-stationary behavior (HSB; reactive line) and low-stationary behavior (LSB; proactive line) lines of zebrafish (*Danio rerio*). These two lines exhibit differences in stress-related behaviors across multiple behavioral assays, learning and memory, glucocorticoid responses, neurotranscriptome profiles, and morphology consistent with the reactive and proactive stress coping styles^5,6,10,11,59,60,62,63^. Therefore, we consider any fish from the HSB or LSB lines to have the reactive or proactive stress coping style, respectively. Lines were generated starting from a wild-caught population from Gaighata in West Bengal, India and were maintained through a bidirectional selective breeding paradigm on behavioral stress response to a novelty stressor^5^. The subjects used in this study underwent 11 generations of this breeding paradigm. Both lines were sexually mature (12–15 months post-fertilization) when testing began. Prior to testing, fish were housed in 40-L mixed-sex tanks all on the same custom-built recirculating system with solid filtration. Fish were kept at a water temperature of 27 °C, on a 14:10 L/D cycle and fed twice daily with Tetramin Tropical Flakes (Tetra, USA). All procedures and experiments were approved by the Institutional Animal Care and Use Committee of the University of Nebraska at Omaha/University of Nebraska Medical Center (17-070-09-FC). All methods were performed in accordance with the relevant guidelines and regulations.

### Pharmacological manipulation

Using a modified protocol for chronic ethanol administration in zebrafish^31^, groups of six fish were housed in a 3-L trapezoidal tank (15.2 height × 27.9 top × 22.5 bottom × 11.4 cm width; Pentair Aquatic Ecosystems) throughout the treatment period. The tank contained either 2-L of 0.75% ethanol (v/v; Sigma-Aldrich) or 2-L of system water as a control over the span of 14 days. Every two days we replaced the entire water in each tank with fresh ethanol or system water. At the end of 14 days, a group of fish was used for either behavioral testing or for quantification of whole-brain GABA_A_R subunit mRNA expression. For behavioral testing we used three groups of six fish for each treatment group, where fish were randomly selected individuals from each of the HSB and LSB lines (36 total fish; *n* = 18 for each treatment group). We used a separate set of tanks to treat a different set of 36 individuals from each line (*n* = 18 for each treatment group) for quantification of GABA_A_R subunit expression. Some fish died during the 14-day treatment period resulting in final sample sizes of 32 individuals from the HSB (*n* = 15 treated, 17 control; *female* = 13, *male* = 19) line and 33 from the LSB (*n* = 16 treated, 17 control; *female* = 14, *male* = 19) that were behaviorally tested. A total of 34 individuals from the HSB (*n* = 17 treated, 17 control; *female* = 15, *male* = 19) line and 35 from the LSB (*n* = 17 treated, 18 control; *female* = 18, *male* = 17) were used for GABA_A_R subunit quantification. In total, ten fish were lost during the treatment period, and we observed no consistent pattern in the timing of mortality. Deceased fish were removed within one day.

To identify a biologically relevant ethanol dose, we conducted a pilot dose–response study. We chronically administered ethanol of varying concentrations and durations to both lines followed by a behavioral stress assay (Novel Tank Diving Test) to measure stress and anxiety-related behaviors. Ethanol treatment began at 0.25% v/v over a period of seven days. We increased both the concentration and duration until an anxiolytic effect was observed in both lines of zebrafish without drug-impaired locomotion (i.e. significant change in depth preference without a significant difference or decrease in distance traveled and stationary time relative to control fish). Due to drug-impaired locomotion at higher ethanol concentrations in the 7-day group and lack of anxiolytic effects at lower concentrations, 0.5% ethanol was the chosen starting point for both the 10- and 14-day groups. Similarly, the 0.5% was the only tested concentration for the 10-day group due to lack of anxiolytic effect. We used total distance traveled and total stationary time during the trials as proxies for locomotion to ensure the chosen concentration of ethanol was not impairing the fish’s ability to swim.

### Behavioral testing

Following the 14th day of treatment, fish designated for behavioral testing were exposed to a novelty stressor by placing them into the Novel Tank Diving Test (NTDT) assay following established procedures^5,10,31,60,99,100^. In brief, fish were netted from their treatment tanks and individually placed in a clear 3-L trapezoidal tank (15.2 height × 27.9 top × 22.5 bottom × 11.4 cm width; Pentair Aquatic Ecosystems) filled with 2-L of system water. We video-recorded the fish for six minutes and quantified behaviors using an automated tracking software (Noldus Ethovision XT Version 14, Wageningen, Netherlands) as previously described^5,6,10,13^. In brief, we used the software to virtually partition the tank into top and bottom halves to measure the number of transitions to the top portion of the tank, time spent in the top portion of the tank (s), total distance traveled (cm), and stationary time (s). We used the entirety of the 6-min trial for analysis. The subject was considered stationary if it was moving less than 0.5 cm/s. Reduced transitions to and time spent in the top half of the tank are indicators of heightened stress and anxiety^5,31,99^. Stationary time and distance traveled were used as proxies for locomotor activity to assess whether or not ethanol treatment impaired general locomotor activity. Testing occurred between 2–11 h after light onset with control and ethanol-treated group testing being randomly distributed. We digitally measured standard length of each fish following completion of the trial.

### Quantification of GABA_A_R subunit expression

We quantified whole-brain expression of four genes that encode for GABA_A_R subunits (*gabra1, gabra2, gadrd,* and *gabrg2* with no known paralogs*;* Supplementary Table S5), and one endogenous reference gene (*ef1a*) using quantitative reverse transcriptase PCR (qRT-PCR) following established protocols^13,60,63^. In brief, whole brains were homogenized with 50–100 µL of zirconium oxide beads (Bullet Blender, Next Advanced) in Tri Reagent (Sigma-Aldrich). Then, we extracted RNA and removed genomic DNA using column filtration (PureLink RNA Mini Kit, Ambion). We subsequently synthesized cDNA using both random hexamers and oligo(dT)_20_ primers (SuperScript IV First-Strand Synthesis System for qRT-PCR (Invitrogen). Finally, we purified the cDNA using Amicon Ultracentrifugal filters (Millipore). We carried out all protocols according to each manufacturers’ protocol.

We ran the qRT-PCR on QuantStudio 7 Flex Real-Time PCR System (Applied Biosystems) using SYBR green detection chemistry (PowerUp SYBR Green Master Mix, Applied Biosystems). For *gabrg2* and *ef1a,* we used primer sequences from previously published studies^84,101^. For the remaining genes, we designed primers using Primer-Blast^102^ with chosen primers either spanning exon-exon junctions or with the amplicon spanning exons where the intron region was over one kilobase (Supplementary Table S5). Primer concentrations were 5 pmol for all genes. Reaction parameters for all genes were as follows: 2 min at 50 °C, 2 min at 95 °C, followed by 40 cycles at 95 °C for 15 s then 60 °C for 1 min. We ran each sample in triplicate. Primers for all genes showed high specificity as evidenced by (1) PCR reaction resulting in a single band on gel electrophoresis, (2) sanger sequencing of PCR amplicon aligned with target gene after using NCBI BLAST and (3) observing a single peak on melt curve analysis following qRT-PCR. We quantified expression using the relative standard curve method and normalized expression to an endogenous reference gene (*ef1a*). *ef1a* expression is stable across sex, tissue types, age, and chemical treatment in zebrafish^101^. For each fish we calculated normalized expression by dividing quantity of gene interest by quantity of *ef1a*. We also checked the validity of *ef1a* as an endogenous reference by comparing its expression between ethanol-treated and control individuals. After normalizing *ef1a* expression by total cDNA input, there was no significant difference in *ef1a* expression between treated and controlled fish [*t*(55) = 1.297, *p* = 0.393].

### Statistical analysis

Six of our nine endpoint measurements were not normally distributed. Five of those six endpoints were still not normally distributed after a log transformation. Thus, we used a generalized linear model (GLZM) applying the identity link function in SPSS (Version 26) to investigate changes in behaviors and normalized gene expressions. The significance value was set at *α* = 0.05. Line (HSB, LSB), sex (male, female) and treatment group (0.75% ethanol, control) were used as between-subject variables. All interaction terms were included in the GLZM model. As the relationship between body size and locomotion is well documented^59,103–105^, we included standard length as a covariate. Since we did not find a significant main effect of sex on behavior [top transitions: Wald *χ*^2^(1) = 2.385, *p* = 0.123; top time: Wald *χ*^2^(1) = 0.852, *p* = 0.356; average top trip: Wald *χ*^2^(1) = 0.179, *p* = 0.672; distance: Wald *χ*^2^(1) = 0.682, *p* = 0.409; and stationary time: Wald *χ*^2^(1) = 0.092, *p* = 0.762] or gene expression [*gabra1:* Wald *χ*^2^(1) = 0.036, *p* = 0.850; *gabra2:* Wald *χ*^2^(1) = 0.382, *p* = 0.536; *gadrd:* Wald *χ*^2^(1) = 1.942, *p* = 0.163; *gabrg2:* Wald *χ*^2^(1) = 1.426, *p* = 0.232], we just used line and treatment group as the only between-subject variables. Since there is only one model for each endpoint, use of goodness of fit criteria to select a model is not applicable. To assess the direction of effects, we investigated the simple main effects within each GLZM. We applied a Benjamini–Hochberg correction to all simple main effect investigations to account for multiple comparisons^106^. To analyze changes in behavior across the trial, we divided the 6-min trial into 1-min bins and ran a repeated-measures generalized estimating equation (GEE) for each behavior with treatment and time (6, 1-min blocks) as factors. We ran separate GEEs for each line and applied an identity link function.

## Supplementary information

Supplementary Information 1.

Supplementary Information 2.

## Data Availability

All data generated or analyzed during this study are included in this published article and its Supplementary Information files.
