# Supplementary material for: Differential effects of ethanol on behavior and GABAA receptor expression in adult zebrafish (Danio rerio) with alternative stress coping styles
Source: Sci Rep. 2020 Aug 4;10:13076. doi: 10.1038/s41598-020-69980-2 (PMC7403336; doi:10.1038/s41598-020-69980-2)
Supplement: Supplementary file 2 — Supplementary Information 2. [file 41598_2020_69980_MOESM2_ESM.pdf]

Supplementary Information

Differential effects of ethanol on behavior and $GABA_A$ receptor expression in adult zebrafish (*Danio rerio)* with alternative stress coping styles

Alexander C Goodman[1] and Ryan Y Wong[1, 2,*]

[1]Department of Biology, University of Nebraska at Omaha, Omaha, Nebraska, USA

[2]Department of Psychology, University of Nebraska at Omaha, Omaha, Nebraska, USA

[*]Correspondence: Ryan Y Wong, University of Nebraska at Omaha, 6001 Dodge St, Omaha, NE 68182 Email: rwong@unomaha.edu Phone: 402-554-4473

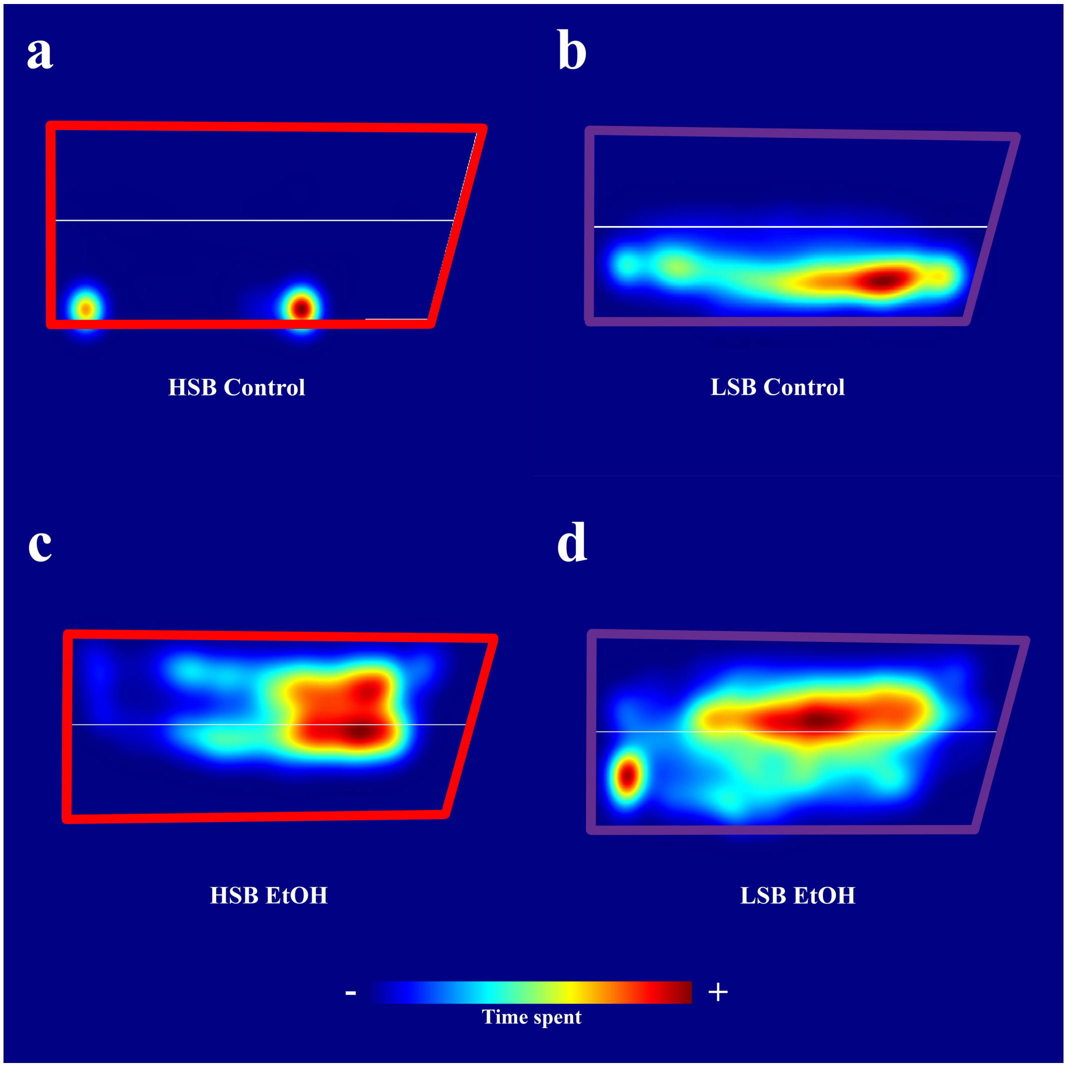

Supplementary Figure S1. Heatmap of exploratory activity during the 6-minute trial of the NTDT for the (**a**) HSB control, (**b**) LSB control, (**c**) HSB EtOH, and (**d**) LSB EtOH groups. Each heatmap is a representative individual that was most similar to the group average. Data was analyzed using video tracking software (Noldus Ethovision XT Version 14, Wageningen, Netherlands).

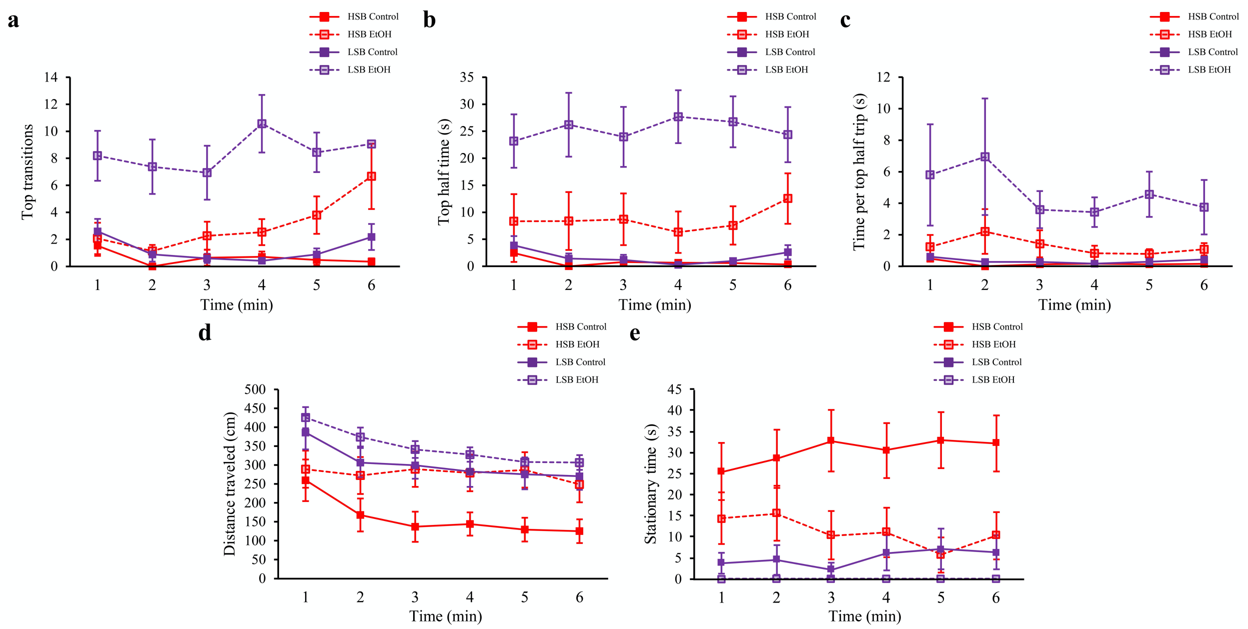

Supplementary Figure S2. Changes in behavior across trial time. We measured top transitions (**a**), time in top half of the tank (**b**), average time spent in top half per trip (**c**), distance traveled (**d**), and stationary time (**e**) for each group. Points represent group means at that minute (± 1 SEM). Control groups are represented by unfilled boxes with sold best-fit lines, while ethanol-treated groups are represented by filled boxes with dashed best-fit lines. HSB and LSB are red and purple, respectively. The number of subjects tested in each group are as follows: 17 HSB control, 17 LSB control, 15 HSB EtOH, 16 LSB EtOH.

Generalized Linear Model (GLZM)							
Treatment Duration	Statistic	Time in Top (s)		Distance Traveled (cm)		Stationary Time (s)	
		HSB	LSB	HSB	LSB	HSB	LSB
7-Day $n_{HSB} = 94$ $n_{LSB} = 11$	$\overline{X}$	33.310	172.730	821.515	2108.263	199.342	0.000
	$\sigma_{\overline{X}}$	9.305	30.534	97.929	199.76	15.921	22.383
	χ^2	**4.405***	**9.736***	**5.103***	2.088	**9.150***	1.502
	p	0.036	0.002	0.024	0.148	0.002	0.22
	B	-32.470	-141.313	-367.817	-428.160	80.078	40.686
	CI (lower, upper)	(-62.793, -2.148)	(-230.079, -52.547)	(-686.960, -48.675)	(-1008.881, 152.561)	(28.191, 131.965)	(-24.384, 105.757)
10-Day $n_{HSB} = 12$ $n_{LSB} = 11$	$\overline{X}$	0.790	78.312	2396.714	2008.400	16.622	23.457
	$\sigma_{\overline{X}}$	0.450	23.376	226.282	198.373	21.723	15.815
	χ^2	0.122	2.640	**41.798***	0.010	**87.957***	0.998
	p	0.727	0.104	1.10×10^{-10}	0.92	< 0.001	0.318
	B	-0.222	-59.330	-2068.925	29.706	288.123	-23.43
	CI (lower, upper)	(-1.470, 1.025)	(-124.285, 11.625)	(-2696.135, -1441.715)	(-546.985, 606.397)	(227.910, 348.336)	(-69.405, 22.544)
14-Day $n_{HSB} = 65$ $n_{LSB} = 46$	$\overline{X}$	67.253	73.404	2294.718	1857.808	33.343	1.768
	$\sigma_{\overline{X}}$	11.617	13.28	132.108	111.403	17.109	15.946
	χ^2	**12.338***	**8.707***	**39.846***	**3.873***	**14.041***	**6.792***
	p	4.43×10^{-4}	0.003	2.75×10^{-10}	0.049	1.79×10^{-4}	0.009
	B	-60.063	-55.415	-1227.495	-310.067	94.365	58.772
	CI (lower, upper)	(-93.577, -26.549)	(-92.224, -18.606)	(-1608.627, -846.364)	(-618.856, -1.278)	(45.006, 143.724)	(14.753, 102.971)

Supplementary Table 1. Summary of all generalized linear models ran for each of the ethanol treatment durations during the pilot dose-response study. Comparisons were made between ethanol treatment groups and control groups for each line at each treatment duration. Abbreviations: HSB, high stationary behavior; LSB, low station behavior; $\overline{X}$, average; $\sigma_{\overline{X}}$, standard error; χ^2, Wald Chi-squared; *p*, p-value; *CI*, 95% Confidence Interval. Wald Chi-square values bolded with a superscript asterisk indicate a significant p-value.

Generalized Linear Model (GLZM)						
Effect	Statistic	Top transitions	Top half time (s)	Time per top half trip (s)	Distance Traveled (cm)	Stationary Time (s)
Intercept	χ^2	**55.176****	**43.928****	**46.712****	**275.650****	**29.558****
	p	1.85×10^{-13}	9.63×10^{-12}	5.61×10^{-12}	< 0.001	6.27×10^{-8}
	B	51.313	151.781	3.264	2101.161	0.000
	CI (lower, upper)	(40.588, 62.037)	(119.236, 184.326)	(2.401, 4.126)	(1710.211, 2492.110)	(-50.430, 50.430)
Strain	χ^2	**12.579***	**10.215***	**5.045***	**11.378***	**18.173***
	p	3.92×10^{-4}	0.001	0.025	0.001	2.02×10^{-5}
	B	-33.446	-99.925	-1.833	-434.801	67.450
	CI (lower, upper)	(-48.863, -18.029)	(-146.711, -53.138)	(-3.073, -0.593)	(-996.826, 127.224)	(-5.048, 139.947)
Treatment	χ^2	**28.054***	**32.659***	**15.227***	**5.792***	**7.831***
	p	1.18×10^{-7}	1.10×10^{-8}	9.53×10^{-5}	0.016	0.005
	B	-42.960	-141.480	-2.557	-243.256	30.008
	CI (lower, upper)	(-57.901, 28.018)	(-186.824, -96.137)	(-3.759, -1.355)	(-787.950, 301.439)	(-40.253, 100.270)
Stain*Treatment	χ^2	**6.788***	**8.182***	3.784	1.391	2.639
	p	0.009	0.004	0.052	0.238	0.104
	B	28.328	94.388	1.701	-467.462	83.069
	CI (lower, upper)	(7.017, 49.639)	(29.714, 159.062)	(-0.013, 3.415)	(-1244.363, 309.437)	(-17.146, 183.284)

Supplementary Table S2. Results of generalized linear model (GLZM) for top transitions, top half time, time per top half trip, distance traveled, and stationary time. Abbreviations: χ^2, Wald Chi-squared; *p*, p-value; *CI*, 95% Confidence Interval. Wald Chi-square values bolded with a superscript asterisk indicate a significant p-value.

Repeated Measures Generalized Estimating Equation (GEE)											
Effect	Statistic	Top transitions		Top half time (s)		Time per top half trip (s)		Distance Traveled (cm)		Stationary Time (s)	
		HSB	LSB	HSB	LSB	HSB	LSB	HSB	LSB	HSB	LSB
Intercept	χ^2	**12.780***	**35.594***	**5.430***	**44.055***	**4.935***	**14.034***	**65.618***	**277.723***	**27.461***	2.733
	p	3.50 x 10^{-4}	2.43 x 10^{-9}	0.020	3.19 x 10^{-11}	0.026	1.80 x 10^{-4}	5.55 x 10^{-16}	< 0.001	1.60 x 10^{-7}	0.098
	B	6.667	9.063	12.543	24.375	1.080	3.748	248.983	306.780	10.332	1.94 x 10^{-16}
	CI (lower, upper)	(2.089, 11.244)	(4.983, 13.142)	(3.697, 21.388)	(14.682, 34.068)	(0.341, 1.820)	(0.465, 7.031)	(158.976, 338.989)	(270.136, 343.422)	(-0.286, 20.950)	(-1.94 x 10^{-16}, -1.94 x 10^{-16})
Treatment	χ^2	**5.664***	**19.532***	3.736	**33.556***	2.808	**10.485***	**4.821***	1.258	**5.711***	2.733
	p	0.017	9.89 x 10^{-6}	0.053	6.92 x 10^{-9}	0.094	0.001	0.028	0.262	0.017	0.098
	B	-6.314	-6.886	-12.189	-21.768	-0.926	-3.319	-123.750	-36.262	21.885	6.249
	CI (lower, upper)	(-10.926, -1.702)	(-11.355, -2.417)	(-21.049, -3.330)	(-31.784, -11.753)	(-1.696, -0.157)	(-6.651, -0.023)	(-231.892, -15.609)	(-133.866, 41.341)	(5.340, 38.430)	(-1.349, 13.848)
Time	χ^2	**14.059***	**14.000***	8.564	1.158	4.469	5.112	**23.359***	**45.354***	2.675	7.083
	p	0.015	0.016	0.128	0.949	0.484	0.402	2.88 x 10^{-4}	1.23 x 10^{-8}	0.750	0.215
Time*Treatment	χ^2	7.061	**12.211***	9.224	10.547	9.231	**13.785***	11.005	2.522	8.084	7.083
	p	0.216	0.032	0.100	0.061	0.100	0.017	0.051	0.773	0.152	0.215
1st minute	*B*	-4.600	-0.875	-4.211	-1.195	0.159	2.049	40.016	118.612	4.028	1.94 x 10-16
	CI (lower, upper)	(-9.057, -0.143)	(-4.646, 2.896)	(-12.345, 3.923)	(-12.613, 10.223)	(-0.869, 1.187)	(-5.110, 9.207)	(-47.066, 127.097)	(78.210, 159.014)	(-7.832, 15.889)	(-1.94 x $10^{-16,}$ - 1.94 x 10^{-16})
2nd minute	*B*	-5.533	-1.688	-4.158	1.829	1.126	3.202	23.525	67.321	5.235	-5.55 x 10^{-17}
	CI (lower, upper)	(-9.685, -1.381)	(-4.785, 1.410)	(-12.814, 4.498)	(-11.880, 15.538)	(-1.083, 3.335)	(-4.679, 11.083)	(-53.734, 100.785)	(27.461, 107.182)	(-4.988, 15.457)	(-5.55 x 10^{-17}, -5.55 x 10^{-17})
3rd minute	*B*	-4.400	-2.125	-3.840	-0.419	0.347	-0.153	40.211	34.447	0.016	-2.77 x 10^{-17}
	CI (lower, upper)	(-8.380, -0.420)	(-4.480, 0.230)	(-9.983, 2.303)	(-13.460, 12.622)	(-0.838, 1.533)	(-4.154, 3.848)	(-22.992, 103.413)	(8.806, 60.088)	(-9.513, 9.545)	(-2.77 x 10^{-17}, -2.77 x 10^{-17})
4th minute	*B*	-4.133	1.500	-6.217	3.318	-0.254	-0.312	30.279	21.086	0.752	-1.11 x 10^{-16}
	CI (lower, upper)	(-7.555, -0.712)	(-0.913, 3.913)	(-12.617, 0.183)	(-7.909, 14.545)	(-0.881, 0.373)	(-3.616, 2.992)	(-23.150, 83.707)	(-14.719, 56.891)	(7.731, 9.235)	-1.11 x 10^{-16}, - 1.11 x 10^{-16}
5th minute	*B*	-2.867	-0.625	-4.986	2.365	-0.294	0.820	38.296	1.326	-4.590	1.11 x 10^{-16}
	CI (lower, upper)	(-5.851, 0.118)	(-2.980, 1.730)	(-9.241, -0.730)	(-4.186, 8.916)	(-0.814, 0.226)	(-1.261, 2.900)	(-3.913, 80.504)	(-24.367, 27.018)	(-10.591, 1.411)	(1.11 x 10^{-16}, 1.11 x 10^{-16})
Treatment* 1st minute	*B*	5.776	1.287	6.335	2.453	0.175	-1.868	94.703	-3.157	-10.801	-2.422
	CI (lower, upper)	(1.049, 10.504)	(-3.173, 5.746)	(-2.419, 15.089)	(-9.491, 14.398)	(-0.953, 1.302)	(-9.036, 5.300)	(-14.537, 203.942)	(-78.415, 72.102)	(-25.560, 3.958)	(-9.270, 4.427)
Treatment* 2nd minute	*B*	5.180	0.393	3.805	-3.001	-1.280	-3.359	19.322	-31.264	-8.890	-1.708
	CI (lower, upper)	(0.990, 9.370)	(-3.114, 3.901)	(-4.866, 12.475)	(-16.972, 10.970)	(-3.499, 0.939)	(-11.252, 4.534)	(-67.926, 106.571)	(-82.790, 20.261)	(-20.930, 3.149)	(-4.318, 0.901)
Treatment* 3rd minute	*B*	4.694	0.537	4.241	-1.006	-0.375	0.002	-36.589	-5.670	-1.365	-4.044
	CI (lower, upper)	(0.558, 8.830)	(-2.521, 3.595)	(-2.020, 10.502)	(-14.453, 12.441)	(-1.562, 0.813)	(-4.029, 4.034)	(-105.468, 32.290)	(-59.437, 48.096)	(11.175, 8.445)	(-9.661, 1.573)
Treatment* 4th minute	*B*	4.486	-3.265	6.520	-5.654	0.268	0.047	-11.423	-8.701	-2.415	-0.057
	CI (lower, upper)	(1.030, 7.942)	(-6.269, -0.260)	(0.108, 12.933)	(-17.161, 5.854)	(-0.371, 0.906)	(-3.270, 3.364)	(-70.456, 47.610)	(-57.327, 39.925)	(-11.478, 6.648)	(-1.559, 1.445)
Treatment* 5th minute	*B*	2.984	-0.669	5.225	-3.992	0.265	-0.959	-34.138	3.648	5.235	0.843
	CI (lower, upper)	(-0.075, 6.043)	(-3.458, 2.119)	(0.882, 9.568)	(-10.924, 2.940)	(-0.257, 0.786)	(-3.047, 1.130)	(-81.200, 12.923)	(-30.993, 38.288)	(-1.986, 12.457)	(-1.770, 3.456)

Supplementary Table S3. Results of repeated measures generalized estimating equation (GEE) for top transitions, top half time, time per top half trip, distance traveled, and stationary time. Abbreviations: χ^2, Wald Chi-squared; *p*, p-value; *CI*, 95% Confidence Interval. Wald Chi-square values bolded with a superscript asterisk indicate a significant p-value.

Generalized Linear Model (GLZM)					
Effect	Statistic	*gabra1*	*gabra2*	*gabrd*	*gabrg2*
Intercept	χ^2	**624.835***	**101.657***	**134.074***	**501.090***
	p	< 0.001	< 0.001	< 0.001	< 0.001
	B	1.748	5.205	4.379	2.052
	CI (lower, upper)	(1.530, 1.966)	(3.693, 6.716)	(3.133, 5.624)	(1.753, 2.351)
Strain	χ^2	**7.310***	**8.235***	0.023	**5.929***
	p	0.007	0.004	0.880	0.015
	B	0.431	-2.006	-0.484	-0.250
	CI (lower, upper)	(-0.743, -0.117)	(-4.177, 0.164)	(-2.273, 1.305)	(0.679, 0.179)
Treatment	χ^2	**6.507***	0.648	2.042	**7.220***
	p	0.011	0.421	0.153	0.007
	B	-0.413	-0.413	-1.489	-0.288
	CI (lower, upper)	(-0.717, -0.109)	(-2.520, 1.695)	(-3.226, 0.248)	(-0.705, 0.128)
Stain*Treatment	χ^2	1.339	0.073	0.832	0.659
	p	0.247	0.787	0.362	0.417
	B	0.258	-0.418	1.160	-0.250
	CI (lower, upper)	(-0.179, 0.694)	(-3.443, 2.608)	(-1.333, 3.653)	(-0.852, 0.353)

Supplementary Table S4. Results of generalized linear model (GLZM) for expression of *gabra1*, *gabra2*, *gabrd*, and *gabrg2*. Abbreviations: χ^2, Wald Chi-squared; *p*, p-value; *CI*, 95% Confidence Interval. Wald Chi-square values bolded with a superscript asterisk indicate a significant p-value.

Gene symbol	Forward Primer	Reverse Primer	Amplicon Length (bp)
ef1a	5'- CCTCTTGGTCGCTTTGC-3'	5'- GGTGTGATTGAGGGAAATTCA-3'	150
gabra1	5'-TGAGTCAGAGACAAGAGTGTTC-3'	5'- CTTCCACCCCACATCATTCTC-3'	107
gabra2	5'- CAGACACTTTCTTTCATAACGG-3'	5'- TCCTCAAGATGCATTGGG-3'	145
gabrd	5'- AACTTTCGTCCAGGGATCGG-3'	5'- TGGTGTATTCCATGTTGGCTTC-3'	100
gabrg2	5'- ACGGCTATGGACCTCTTCGT -3'	5'- TTTGAGGAAAAGAGCCGCAGG -3'	155

Supplementary Table S5. qRT-PCR primer characteristics.
